# Editorial: Crop Response to Waterlogging

**DOI:** 10.3389/fpls.2019.01578

**Published:** 2019-12-11

**Authors:** Iduna Arduini, Makie Kokubun, Francesco Licausi

**Affiliations:** ^1^Department of Agriculture, Food and Environment, University of Pisa, Pisa, Italy; ^2^Tohoku University, Sendai, Japan; ^3^Department of Biology, University of Pisa, Pisa, Italy

**Keywords:** climate change, crop species, flooding, multidisciplinary approach, waterlogging

Soil waterlogging occurs when excess water saturates the soil pores with either no layer of water or a very fine one on the soil surface. Since gas diffusion in water is several folds slower than in air, the oxygen concentration decreases rapidly in waterlogged soils, thereby triggering a cascade of conditions that are detrimental to the growth of most plant species (Colmer and Greenway, 2011).

Flooding is the primary cause of yield losses in Asia and Latin America while, worldwide, between 10 and 16% of the arable soils are estimated to be affected by waterlogging (Yaduvanshi et al., 2014; FAO, 2015). Moreover, the frequency and intensity of flooding events is expected to increase in all world regions in consequence of climate change (Westra et al., 2014; IPCC, 2018). Thus, the purpose of the research topic “Crop Response to Waterlogging” is to gather knowledge from different geographic regions and research fields in order to help the scientific and social community to elaborate an integrated approach to face its adverse effects.

The articles published in this research topic deal with a wide range of crop species, including cereals (Arduini et al.; Panozzo et al.; Yamauchi et al.), forage and grain legumes (Pucciariello et al.; Zhang et al.), and tree crops (Ruperti et al.; Iacona et al.), thus demonstrating the widespread interest in plant sciences for this soils condition. The researches published in this e-book describe different investigation approaches and propose alternative solutions, which range from the reduction of waterlogging risk by improving agronomic and engineering solutions (Manik et al.; Zhang et al.), to the better understanding of crop response to waterlogging (Arduini et al.; Ejiri and Shiono; Lin and Sauter; Pucciariello et al.; Ruperti et al.; Yamauchi et al.), and the selection of crops and genotypes that are more tolerant to waterlogging conditions (Iacona et al.; Panozzo et al.; Pompeiano et al.).

Waterlogging generally induces changes in gene expressions, which result in physiological and morphological changes in plants. Flooding responses on grapevine are reviewed by Ruperti et al., and their findings enable us to draft a first comprehensive view of the metabolic pathways involved in grapevine’s root responses and to highlight a transcriptional and metabolic reprograming during and after exposure to waterlogging. The gene expressions of *AOX1A*, *CYP81D8*, and putative *PFP* genes were analyzed in a large set of commercial maize hybrids under extreme waterlogging conditions and their involvement in the morphological changes are discussed (Panozzo et al.).

Roots are the first target of waterlogging stress in plants, and it is well documented that aerenchyma formation plays a crucial role in internal long-distance oxygen transport from shoot to root under waterlogged soil. A comparison of the cross-sectional area of root tissues among three crop species (wheat, maize, and rice), subjected to aerated or deoxygenated conditions, revealed that high cortex to stele ratio in combination with large root diameter is a feature which promotes oxygen transport from shoot to root tips (Yamauchi et al.). Previous studies indicate that a structural barrier is formed in wetland plant root, which might help impede radial oxygen loss in the course of oxygen transport. Three species of *Echinochloa*, well adapted to various soil water conditions, were found to constitutively develop the barrier under aerated conditions, and suberin was an important component of the barrier (Ejiri and Shiono). These studies provided new findings on the morphological features of waterlogging-adapted plant species. Moreover, Lin and Sauter found that auxin signaling is likely to contribute to the coordinated processes of adventitious roots formation, which might support or replace the main root system in flooded rice plants.

Flooding-induced physiological dysfunctions include the impairment of water and nutrients uptake, of hormonal balance and photosynthesis, which consequently result in poor growth under flooding. In legume crops, flooding conditions can harm N assimilation through weakened capacity for symbiotic N_2_ fixation. The mechanisms underlying symbiotic processes and functioning under waterlogging are reviewed, with a focus on the mechanisms of oxygen sensing of the host plant and the symbiotic partner (Pucciariello et al.). The findings presented in this review would help in understanding the mechanisms regulating flooding tolerance in legume species, which may lead to isolate tolerant crop species and varieties in the field. The severity of yield reduction by waterlogging varies with crop species, partly because of the specific phenology of each of them. In the Mediterranean environment, winter cereals can experience waterlogging at their early growth stage. Late sown oats subjected to waterlogging at tillering failed to produce acceptable yield, primarily because of insufficient capacity for tiller and panicle formation after the release from waterlogging (Arduini et al.).

Under severe waterlogging, in addition to genetic improvement, agronomic and engineering measures are required to minimize the impact of waterlogging. Hence, management practices to alleviate the damage by waterlogging are reviewed (Manik et al.). Based on their comprehensive analyses of literature on crop management practices, the authors suggest that the strategies to withstand waterlogging should be adopted in a manner where genetic, agronomic, and engineering measures are complementarily integrated depending on the respective environments. They suggest further research aspects such as the comparison of cost/benefit analyses of different drainage strategies, understanding the mechanisms of nutrient loss during waterlogging and quantifying the benefits of nutrient application. Among strategies to adopt, Zhang et al. showed evidence that melatonin improves waterlogging tolerance in alfalfa by reprogramming polyamine and ethylene metabolism. In addition, photosynthetic performance and growth response of giant cane, which spontaneously grows in different kinds of harsh environments, are characterized, with the aim to replace sensitive crops in waterlogging prone soils (Pompeiano et al.).

The research published in this topic clearly demonstrate that a multidisciplinary approach is crucial to reduce waterlogging risk and ameliorate its adverse effects on crops. The first step should be the adoption of soil management practices that reduce soil compaction and improve drainage (Manik et al.), followed by the use of crop management techniques that increase plant resistance (Zhang et al.), or the choice of crops and genotypes that are more adaptable to soil hypoxia (Pompeiano et al.; Iacona et al.; Panozzo et al.). The published articles also highlight that crop species respond differently to waterlogging stress, so that basic research is still needed.

To conclude, we are deeply grateful to all scientists who answered to the call and allowed us to publish their researches in this collection on “Crop Response to Waterlogging,” which provides the unique opportunity to integrate the knowledge from many research fields for facing waterlogging and reduce crop loss worldwide.

## Author Contributions

Authors contributed equally to writing and revising the editorial.

## Conflict of Interest

The authors declare that the research was conducted in the absence of any commercial or financial relationships that could be construed as a potential conflict of interest.
